# New Insights into Mechanisms of Endocrine-Disrupting Chemicals in Thyroid Diseases: The Epigenetic Way

**DOI:** 10.3390/ijerph17217787

**Published:** 2020-10-24

**Authors:** Letizia Pitto, Francesca Gorini, Fabrizio Bianchi, Elena Guzzolino

**Affiliations:** 1 Institute of Clinical Physiology, National Research Council, 56124 Pisa, Italy; fgorini@ifc.cnr.it (F.G.); fabriepi@ifc.cnr.it (F.B.); elena.guzzolino@gmail.com (E.G.); 2 Department of Biosciences, University of Milan, 20133 Milan, Italy

**Keywords:** thyroid, endocrine disrupting compounds, epigenetics, environmental pollution, exposure

## Abstract

In recent years, the presence in the environment of chemical compounds with thyroid-disrupting effects is progressively increased. This phenomenon has risen concern for human health as the preservation of thyroid system homeostasis is essential for fetal development and for maintaining psychological and physiological wellbeing. An increasing number of studies explored the role of different classes of toxicants in the occurrence and severity of thyroid diseases, but large epidemiological studies are limited and only a few animal or in vitro studies have attempted to identify the mechanisms of chemical action. Recently, epigenetic changes such as alteration of methylation status or modification of non-coding RNAs have been suggested as correlated to possible deleterious effects leading to different thyroid disorders in susceptible individuals. This review aims to analyze the epigenetic alterations putatively induced by chemical exposures and involved in the onset of frequent thyroid diseases such as thyroid cancer, autoimmune thyroiditis and disruption of fetal thyroid homeostasis.

## 1. Introduction

A continuous release of chemicals into the environment represents a growing risk for all living organisms exposed in different ways to an ever-increasing variety of products. Numerous chemicals are suspected to be associated with various, and often severe, health problems in humans. A class of compounds that have more recently raised concern in society as a possible serious threat to health are the endocrine-disrupting chemicals (EDCs). An endocrine-disrupting compound has been defined by the U.S. Environmental Protection Agency (EPA) as “an exogenous agent that interferes with synthesis, secretion, transport, metabolism, binding action, or elimination of natural blood-borne hormones that are present in the body and are responsible for homeostasis, reproduction, and developmental process.” [1]. EDCs include a wide variety of chemicals such as drugs, pesticides, plastic softeners, flame retardants and represent a significant component of the environment, food, and consumer product contaminants [1,2]. 

Some EDCs are believed to specifically exert thyroid-disrupting effects [3]. Thyroid hormones (THs) have pleiotropic effects and a central role as regulators of metabolism, bone formation, myocardial contractility and neural growth and differentiation [4,5,6,7]. Moreover, THs are of special importance for fetal growth and differentiation [8,9]. Therefore, substances with disrupting effects for thyroid homeostasis represent a potential hazard to public health and the comprehension of the mechanisms through which they can produce their negative effects is of outmost importance. In recent years, several experimental studies and more limited epidemiological evidence indicate epigenetic changes induced by EDC exposure as a possible underlying mechanism of action of these compounds [10]. Epigenetics, i.e., gene function alterations that are heritable but reversible and do not entail a modification in nucleotide sequence, is a general term defining different processes including DNA methylation, histone tail modification, and non-coding RNAs [11]. All mechanisms are strictly related as DNA methylation affects histone modifications and vice versa, while non-coding RNAs, microRNAs and long non-coding RNAs strongly contribute to the regulation of gene expression as well as in shaping chromatin structures [11,12]. The importance of epigenetic control was dramatically highlighted with the advent of high-throughput genomic technologies which revealed that: (i) only <3% of the mammalian genome is transcribed into protein-coding mRNAs and, even more importantly, (ii) the proportion of non-protein-coding DNA increases with and is responsible of developmental complexity raise [13]. Since a complicated network of epigenetic elements and transcription factors, which in turn modify epigenetic components, are at the root of physiological processes, it is not inconceivable that EDCs (known to interfere with the hormonal system via various pathways), may affect also and even with more probability the epigenetic machinery. Although the epigenetic action of EDCs is now widely recognized, studies that allow an EDC-induced thyroid pathological state to be directly associated with a specific epigenetic modification, are extremely rare. In this review we will focus on three main classes of thyroid disorders that can be associated to EDC exposure: disruption of thyroid hormone balance in utero, thyroid cancers and autoimmune thyroiditis, and we will attempt to show the possible role played by epigenetic mechanisms in the relationship between exposure to toxic compounds and onset and/or progression of thyroid disease.

## 2. Thyroid Hormone Balance Alteration Following Prenatal Exposure 

THs are essential for normal growth and development of the fetus. The fetus relies exclusively on maternal THs [14,15], since the onset of thyroid function in the human fetus occurs only around 16–20 weeks of gestation, while the fetal thyroid gland reaches full maturation only towards the end of gestation [16,17]. Hence, active transport of maternal THs across the placenta occurs during this early period of gestation [18], and a significant transfer of TH from the mother to the fetus also persists during the second half of gestation [19]. Thyroxine (T4) is the primary TH to be transferred across the placenta and, although TH total levels in fetal fluids are more than 100-fold lower than those in the maternal compartment, the unbound fraction is much higher in comparison [20]. Moreover, T4 and free T4 levels in the fetus steadily increase and reach adult levels at the beginning of the third trimester of gestation, whereas T3 and free T3 levels remain low during fetal life, showing an increase only at term [21].

Thus, maternal exposure to compounds with thyroid-disrupting properties during pregnancy can affect not only the health of the mother but also the child before and after delivery, possibly resulting in fetal disease. Indeed, even small changes in TH levels of pregnant woman were shown to cause deleterious effects in the fetus [22,23,24,25,26]. Moreover, a growing number of studies demonstrated TH level alterations in pregnant women and in newborn infants following exposure to thyroid-disruptors such as organochlorine pesticides, polybrominated diphenyl ethers (PBDEs) or polychlorinated biphenyls (PCBs) [27], although some of the observed associations were inconsistent [22,28,29,30,31]. However, so far, the mechanisms explaining such mother–child pair relationships remain almost unknown. A recent paper suggested that epigenetic mechanisms could contribute to xenobiotic dysregulation of thyroid homeostasis in mother and fetus [32]. Starting from the observation that epigenetic alterations in placental tissues were associated to maternal exposure to EDCs, the authors investigated the possible associations between prenatal exposure to some persistent organic pollutants (POPs) and DNA methylation of three genes coding for proteins that are crucial for placental TH supply: (i) deiodinase type 3 (DIO3), the most abundant deiodinase in villous trophoblast [33], which plays a central role in protecting the fetus from excessive TH exposure; (ii) monocarboxylate transporter 8 (MCT8), a TH transporter of the syncytiotrophoblast, which facilitates thyroxine (T4) and triiodothyronine (T3) uptake directly from the maternal circulation in vivo; and (iii) transthyretin (TTR), an important TH carrier that is taken up by trophoblasts and translocated to the fetal circulation, forming a TTR shuttle system [34]. The authors found a positive and significant correlation between p,p-dichlorodiphenyldichloro ethylene and BDE-47 serum concentrations and placental DIO3 methylation among female infants, while in males exclusively p,p′-dichlorodiphenyltrichloroethane serum concentration was positively correlated with MCT8 methylation. At the moment, the dimorphism observed in the methylation of DIO3 and MCT8 has no explanation, albeit sex-specific associations between exposure to POPs and global methylation was already reported [35,36,37]. Conversely, the placental TTR gene did not undergo methylation but the ability of hydroxylated metabolites of PCBs and PBDEs to interact with TTR was reported in in vivo and in vitro investigations [38,39,40]. These results were further strengthened by a previous research, performed in the same cohort, which showed a significant negative relationship between cord serum or maternal serum concentrations of POPs and TH level in newborns, whereas a significant positive association was observed with TSH bloodspot [27]. 

Although Kim’s paper observed an alteration of methylation status for only two placental genes, a deep modification of methylation patterns in placenta is probably an important and wider effect at least as a consequence of POP exposure. Accordingly, decreased methylation of long interspersed element-1 (LINE-1, considered a marker of methylation level) and increased methylation of imprinted IGF2 and H19 (codifying for a long non-coding RNA, see chapter 3) genes in placenta were found after prenatal exposure to POPs [38].

## 3. Thyroid Cancer

Thyroid cancer (TC) is the most frequently diagnosed malignancy of the endocrine system [39]. Thyroid neoplastic transformation can occur in either the follicular or parafollicular cells of the gland and can be classified on the basis of their differentiation status. Well-differentiated thyroid cancers are papillary thyroid carcinomas (PTCs), follicular thyroid carcinomas (FTCs), and Hürthle cell carcinomas, which are the most frequent thyroid neoplasms with an overall 5-year survival of approximately 85–90% of cases [40,41]. On the other hand, higher lethality characterizes poorly differentiated thyroid carcinomas and, in particular, the anaplastic thyroid carcinomas (ATCs), representing one of the most lethal cancers in humans [42]. A worldwide steady rise of thyroid tumors (prevalently as PTCs) has been reported over the past several decades [43]. Although an increase in the use of more sensitive imaging techniques such as CT scans, which are able to identify even small thyroid nodules, can have contributed to the dramatic increase of thyroid tumor incidence, environmental chemicals are also believed to play a crucial role in this phenomenon. 

Carcinogenicity of certain chemical compounds is well established. However, there is a paucity of epidemiological evidence on the relationship between exposure to environmental contaminants and risk of thyroid cancer due to the reduced number of extensive epidemiological studies exploring the outcomes following exposure to chemicals whose negative effects on thyroid function were experimentally reported [44]. The only compounds widely investigated are PCBs albeit their correlation with risk of thyroid cancer are still conflicting [45,46]. A few data suggested a positive association of high levels of cadmium (Cd) in thyroid tissue with advanced stage of thyroid cancer [47,48]. More controversial data were instead reported for lead exposure [49]. Flame retardants such as PBDEs were reported to increase thyroid follicular adenomas in animal models, however, studies in humans did not generally support a positive association between PBDE exposure and occurrence of thyroid tumors [50,51]. Bisphenol A (BPA) was associated with increased risk of thyroid nodules (TNs) in Chinese women [52], in accordance with experimental research on animals that strengthened an involvement of this toxicant in thyroid cancer [3]. Additionally, BPA substitutes and halogenated derivatives of BPA do not emerge as safer alternatives to BPA in term of TH disruption and may alter thyroid function at different levels in several cell lines, while in humans some evidence suggested the potential of bisphenols in increasing the risk of TNs [53]. A higher risk of thyroid carcinomas was particularly observed in different volcanic areas of the world, such as Hawaii, Iceland, French Polynesia, New Caledonia, and Sicily in Italy [54,55]. The increased content of heavy metals in soil and, consequently, in plants grown in these areas, as well as atmosphere pollutants (CO_2_, sulfur, and chlorine compounds) or other potential thyroid disruptors contained in waters such as fluorine, sulfur, and selenium, could be responsible for the higher incidence of thyroid tumors in volcanic areas. Another widespread contaminant of drinking water is nitrate largely used as fertilized and therefore particularly abundant in agricultural areas. Several studies reported the association between nitrate ingestion and increase of thyroid tumor risk [56,57]. As the molecular mechanisms leading to the different types of thyroid tumors are still largely unknown, the causative relationships between thyroid tumors and the exposure to a specific toxicant are difficult to identify [55]. 

It has been hypothesized that thyroid homeostasis disruption could be at the base of the carcinogenesis process triggered by the environmental pollutants [58]. The structural homology of some toxicants, i.e., PBDEs or BPA, to THs suggests that they competitively bind to TH receptors, which results in reduced TH circulation and consequent abnormal proliferation of thyroid tissue eventually ending in tumor [59,60]. A number of compounds can form chromosomal adducts that are potentially mutagenic and cause chromosomal aberrations. In particular, a RET/PTC1 rearrangement, probably caused by DNA breakage at chromosomal fragile sites, was frequently observed in PTCs [61]. Pesticides, nitrates and benzene were reported to increase susceptibility to chromosome breakages [62,63,64]. Nitrate, in particular, might exert its carcinogenic effects through the overproduction of nitric oxide (NO), which is known to promote genomic instability. NO levels are increased in condition of blood hypoxia potentially induced by overproduction of nitrite caused by excessive nitrates uptake [56]. It has been suggested that nitrate might also act as carcinogen potentially affecting thyroid function. In fact, nitrate is able to interfere with the uptake of iodide by the thyroid and, therefore, with the production of THs. TH reduction causes a compensatory increase of TSH, which in turn can induce hypertrophy and thyroid diseases including carcinoma, at least according to studies in animal models. Against this hypothesis is the observation that in many of the association studies between nitrate ingestion and thyroid tumor risk increase, an association between nitrate and hypothyroidisms has not been demonstrated [56].

Exploiting a completely different mechanism, thyroid-disrupting compounds such as PBDEs and polycyclic aromatic hydrocarbons (PAHs), can promote tumor development and progression by upregulating the cytochrome-P450 enzymes, which leads to the production of elevated levels of reactive oxygen species (ROS) and oxidative stress, and in turn can foster tumorigenic processes [65,66,67,68]. 

More recently, epigenetic mechanisms were suggested to have a critical action in thyroid tumorigenesis. As for the most part of tumors, also thyroid tumors exhibit DNA methylation alterations. The first DNA methylation studies in thyroid cancer assessed the DNA methylation status of promoter regions of specific candidate genes. By this approach, aberrant methylation was identified for PTEN (phosphatase and tension homologue), a fundamental tumor suppressor gene [69], and BRAF (B-Raf proto-oncogene, serine/threonine kinase), an oncogene whose mutations are frequently associated to thyroid cancer [70]. Successively, several array-based studies using different platforms have been performed to analyze the methylation profiles of tissue samples from patients with thyroid cancer [71,72]. These studies highlighted that PTC has one of the lowest frequency of DNA methylation alterations [73], while ATC exhibits a frequency of DNA methylation alterations which was 10 fold that of PTC [74] 

Nitrate exposition with the consequent NO overproduction could represent of the causes of alterations in methylation levels: NO treatment on cancer cell lines was shown to induce alteration of methylation patterns although the epigenetic effects through DNA methylation were diverse and contradictory in different lines [75]. A recent paper [76] suggested that NO may interfere with methylation patterns by inhibiting the different isoforms of ten-eleven translocation (TET) enzymes, which are responsible of the active removal of methyl group via the catalysis of 5-methylcytosine (5mC) oxidation to 5-hydroxy- (5hmC), 5-formly, and 5-carboxy [77,78]. 

Moreover, NO can affect another class of epigenetic modifications that were found to be associated with the insurgence and aggressiveness of tumors: modifications of the histone tails [79,80,81]. Alteration of histone tail modification patterns (in particular acetylation and methylation of histone tails) has a substantial impact on cell proliferation and, for this reason, the enzyme that regulate these modifications was proposed as a potential prognostic marker for several tumor types [82,83]. The importance of these modifications was demonstrated also in differentiated thyroid neoplasms wherein, for instance, the Thyroid Transcription Factor-1, essential for thyroid organogenesis and regulating various thyroid-specific genes, was silenced by hyperacetylation on H3-lys9 and increased methylation [84]. NO was shown to be involved in the regulation of posttranscriptional modification of histone tails by directly inhibiting the histone demethylase KDM3A activity as further supported by the significant increase in dimethyl Lys-9 on histone 3 observed in cancer cells exposed to NO [85]. Overall, these data are suggestive of an important role of NO as epigenetic regulator although it has not been reported so far that these epigenetic mechanisms exerted by NO act on thyroid tumors. 

Important epigenetic players in thyroid tumorigenesis processes are also microRNAs (miRNAs). miRNAs are small endogenous RNA sequences able to post-transcriptionally regulate gene expression and involved in the modulation of the vast majority of cellular processes, namely proliferation, development, differentiation and even tumorigenesis [86]. Moreover, miRNAs can behave as either oncogenes or tumor suppressors under certain conditions and a large body of evidence has reinforced the possibility that miRNAs act both as potential biomarkers for human cancer diagnosis and prognosis and possible new therapeutic targets or drugs [87]. 

In TC, a decrease in the expression of DICER, a crucial player in miRNA biosynthesis, was associated with the occurrence of malignant thyroid tissues, as compared with normal thyroid tissues and benign thyroid lesions. Decreased DICER expression was also correlated with important features suggestive of tumor aggressiveness such as the presence of the BRAFV600E mutation and of the RET/PTC1 rearrangement [88,89]. Furthermore, miRNA signatures may be used as prognostic biomarkers of neoplastic lesions and help to understand the causative mechanism of thyroid carcinogenesis [90,91,92]. Specifically, miR-221 and miR-222, belonging to the same miRNA family, were consistently upregulated in PTC as well as in ATC. Thyroid tumors with a marked miR-221/222 upregulation showed a dramatic decrease of the proto oncogene Kit that controls a series of important cellular processes (e.g., cell growth and proliferation, survival, and migration). In PTCs, miR-221 and miR-222 also negatively affected the expression of CDKN1B (p27Kip1), a member of the cyclin-dependent kinase (CDK) inhibitors that are important cell cycle regulators [91]. Reduction or absence of p27Kip1 was frequently associated with ATC as compared with PTC or FTC. More recently, miR-222 was shown to contribute to PTC development by targeting PTEN (phosphatase and tension homologue), a fundamental tumor suppressor gene [93]. Besides miR-221/222, miR-146, which is consistently upregulated in PTC, FTC and ATC, was reported to be involved in the regulation of the Kit oncogene. MiR-146 expression positively was correlated with oncogenic potential and reduced susceptibility to chemotherapeutic drug-induced apoptosis in PTC patients and PTC-derived cell line, suggesting a causative role of this miRNA in thyroid tumors [94]. Overall, miR-146, miR-221 and miR-222 displayed the highest diagnostic accuracy in differentiating between malignant and benign thyroid nodules [95].

miR-181, a miRNA characterized as oncogene in prostate, ovary, and stomach cancers, was further identified as upregulated in thyroid neoplastic lesions in several investigations [96,97]. In particular, miR-181actively promoted thyroid tumor growth by targeting the tumor suppressor RB1 [98]. 

Additionally, miRNAs were observed to be responsive to numerous environmental agents, many of which are also known for their action as EDCs. BPA and metal-rich particulate matter determined a higher miR-222 amount, while miR-146 levels were increased by BPA and reduced by Cd [99,100,101]. An increase of miR-181 expression was also observed to be associated with dioxin exposure. Toxicants can also modulate miR-146 level by influencing the expression of the nuclear factor -kappa B (NF-kB), a transcription factor that is activated in human ATC and directly controls miR-146 expression [102]. Oxidative stress induced by an excessive production of ROS as a consequence of exposure to toxic metals appears as one of the most probable cause of NF-κB activation [103]. It is worth noting that oxidative stress is generally considered one of the main reason of miRNA alterations subsequent to the exposure to environmental chemicals [104]. Therefore, changes in expression of miRNAs by toxicant exposure could contribute to thyroid carcinogenesis.

There is another class of non-coding RNAs whose deregulation has recently been demonstrated to participate in cancer progression and is increasingly considered as an important toxicological response to xenobiotics: the long non-coding RNAs (lncRNAs) [105]. lncRNAs can regulate biological processes by controlling numerous cellular functions: nuclear architecture, chromatin structure and transcription in the nucleus as well as mRNA stability, translation and post-translational events in the cytoplasm can be modified by lncRNA activity [106]. Moreover, accumulating evidence indicated the possibility that a crosstalk between lncRNAs and microRNAs may cause tumor aggressiveness and metastasis [107]. A number of lncRNAs were deregulated in thyroid tumors [108] but, at present, only for some of them an alteration was proven as a result of exposure to toxic substances.

The Metastasis-Associated Lung Adenocarcinoma Transcript 1 (MALAT1) is a lncRNA overexpressed in different forms of thyroid carcinoma and its repression in MTC-derived cell line negatively affected cell proliferation and invasion [109,110]. Furthermore, MALAT1 was established to promote epithelial mesenchymal transition (EMT) by interacting with Ezh2, a member of the polycomb chromatine repressive complex2 (PRC2), to silence E-cadherin expression [111].

As for MALAT1, also the Homeobox transcript antisense RNA (HOTAIR) lncRNA is upregulated in human TC cells. HOTAIR overexpression correlated with metastasis and poor prognosis of TC patients [112], whereas in vitro silencing of this lncRNA significantly inhibited cell growth and invasion through mechanisms depending on interaction with PCR2 [113]. In coke oven workers exposed to PAHs [114], the expression levels of both MALAT1 and HOTAIR in peripheral blood lymphocytes were positively associated with the concentration of urinary PAH metabolites and the degree of DNA damage induced by PAHs. A direct relationship between the overexpression of lncRNAs and environmental-induced thyroid tumors has not yet been reported; however, the DNA damages resulted by the PAHs-induced dysregulation of these molecules could potentially generate neoplastic lesions in different body districts.

By contrast with MALAT1 and HOTAIR, the lncRNA Growth Arrest-Specific 5 (Gas5) was significantly downregulated in both PTC tissues and PTC cell lines [93,115]. A low expression of GAS5 characterized several different kind of tumor [116] and was associated with poor prognosis in patients with thyroid cancer [115]. Indeed, GAS5 can operate as tumor suppressor [117]. In thyroid cells GAS5 was shown to act as competing endogenous RNA (ceRNA) by sequestering (“sponging”) miR-222 [71]. Therefore, the substantial downregulation of GAS5 in PTC could be one of the causes of the observed miR-222 upregulation with consequent repression of the PTEN/AKT pathway and increase of the proliferative capacity of thyroid tissue. Nonetheless, there are currently no data concerning GAS5 alteration following exposure to EDCs.

Finally, H19 is a characteristic lncRNA able to act as oncogene in several cancer types and exhibiting a higher expression both in thyroid tumors and in TC cells [118,119]. In thyroid cancer, H19 may exert its oncogenic action by interacting with miR-17-5p and antagonizing its normal function. This interaction resulted in the upregulation of the miR-17-5p target Yamaguchi sarcoma viral oncogene homolog 1 (YES1) with subsequent promotion of cell proliferation and inhibition of apoptosis [120]. As already mentioned in the previous chapter, H19 expression may be affected by POP exposure, at least in the placental tissues [38].

## 4. Autoimmune Thyroiditis

Hashimoto and Graves’ diseases (respectively HT and GD) are the most common forms of autoimmune thyroid disease (AITD), and are characterized by an inflammatory state of the thyroid gland with lymphocytic infiltration and auto reactivity against thyroglobulin, thyroid peroxidase and thyroid stimulating hormone receptor (TSH-R) [121]. Even though GD and other autoimmune thyroiditis can be considered the archetypes of organ-specific autoimmunity, their etiology is still not completely elucidated [122]. In particular, epidemiological evidence has indicated that genetic and environmental factors are involved in the AITD pathogenesis but their relative contribution is still under debate [123,124,125,126]. The importance of genetic susceptibility to these diseases was demonstrated by studies on twin cohorts that observed a concordance rate for Graves’ disease [127], and linkage studies that identified polymorphisms in several loci including genes for human leukocyte antigen, cytotoxic T lymphocyte antigen-4, protein tyrosine phosphatase nonreceptor-type 22, thyroglobulin, vitamin D receptor, and cytokines immune-modifying genes [128].

Among environmental factors able to trigger autoimmune thyroiditis in susceptible individuals, influencing the incidence or the progression of the pathologies, excessive dietary iodide, selenium deficiency, stress, certain drugs, some infectious diseases are considered to be of utmost importance [129]. In addition, exposure to pollutants can result in thyroid autoimmunity, as reported both in experimental animals and in humans [130,131]. 

People resident in areas surrounding a petrochemical complex displayed a higher risk to develop HT and increased level of anti-thyroid antibodies compared to residents in a control area [132]. Signs of autoimmune thyroiditis such as hypoecogenicity (HYE), higher serum level of thyroid peroxidase antibodies (TPO-Ab) and TSH were observed in individuals exposed to PCBs, pesticides and dioxin [133,134] 

A higher prevalence of primary hypothyroidism, anti-microsomal thyroid and anti-thyroglobulin antibodies (Tg-Ab) were reported in workers from a factory producing polyhalogenated biphenyls (PBB) and PBB oxides [135]. Moreover, administration of potassium bromide as a surrogate for PBB, to NOD.H2h4 mice (rodents with a genetic predisposition to autoimmune thyroiditis), increased the likelihood of these animals developing the pathology [130]. 

A limited number of epidemiological studies correlated PBDE exposure with the presence of markers of a subclinical stage of AITD. A significant association between TPO-Ab and BDE-209 was found among women in a population of workers in a deca-BDE manufacturing plant [136]. Although a significant BDE-209/thyroid antibodies ratio in the whole population was not detected, an increase in Tg-Ab and T4 level was correlated with PBDEs in adult male sport fish consumers [137]. Lastly, a 2017 study on a sample including 5628 randomly selected Chinese adults, demonstrated for the first time a relationship between the blood levels of heavy metals and thyroid antibodies [138]. A positive correlation was observed in women between lead, TSH and TPO-Ab as well as between Cd and Tg-Ab, while in men a correlation was detected exclusively between Cd and the highest tertile of Tg-Ab concentration. This study, like many others in this field [132,134,139,140], highlighted significant sex differences in susceptibility to AITDs, observation in line with the ten times higher probability to develop AITD for women compared to men [122].

Overall, these results firmly supported the contribution of environmental pollutants to thyroid autoimmunity, albeit their mechanisms of action are yet to be fully established. Epigenetic mechanisms may exert an important role in AITD. The involvement of epigenetic mechanisms and, in particular, of methylation status alteration, in autoimmune diseases has been acknowledged since the end of the last century when chemical inhibition of DNA methylation in mouse CD4+ T cells was shown to induce the presentation of self-antigens and cause a lupus-like syndrome [141]. A growing body of evidence successively confirmed this observation sustaining that epigenetic modifications are important determinants in several autoimmune diseases including AITD [142]. Changes in DNA methylation represent one of the epigenetic mechanisms more extensively analyzed in AITD. In fact, AITD susceptibility, HT severity and GD tractability were associated to polymorphisms of different components of the DNA methylation pathway: in DNA methyltransferases (DNMTs), the enzyme responsible of the addition of a methyl group to the DNA, but also in genes required for the production of S-adenosylmethionine, the intracellular donor of methyl groups (methylenetetrahydrofolate reductase, and methionine synthase reductase) [143,144]. More recently, high-throughput analysis in GD patients showed GD-distinctive DNA methylation patterns. Significantly decreased transcription of DNMTs and MeCP2 (a protein that binds and recruits different chromatin factors to methylated DNA sequences) was observed in GD patients as compared with normal controls [145]. Furthermore, an elevated number of hypermethylated and hypomethylated regions were identified in AITD patients. Specifically, hypermethylated CpG sites of genes involved in T cell signaling were detected in CD4+ and CD8+ T cells of GD patients [146]. 

Besides the effects of EDCs on methylation of placental tissues, studies carried out mainly on animal models showed that EDCs may interfere at the global body level on methylation affecting both DNMT expression and on the level of ten-eleven translocation methyl cytosine dioxygenase (TET) that is the enzyme responsible of the active removal of methyl group [77,78]. The oxidative stress induced by several environmental factors could further cause oxidation of cytosine at CpG regulatory sites by disrupting the binding of transcriptional regulators to these sequences and inducing irregularly active demethylation via TET [147]. Moreover, BPA can affect DNMT expression level by increasing miRNA-29, which targets DNMTs and reduces the nuclear localization of TET2 in vivo [148]. In humans, PCBs were associated with alteration of global methylation in Greenland Inuit [149] or Korean [37,150] populations, although with conflicting results [37,149,150]. Conversely, no studies experimentally proved changes in methylation status as mechanism of EDC action in AITD. 

During the last few years an impressive number of microRNAs have been identified as differentially expressed in thyroid tissue, serum and peripheral blood mononuclear cells of AITD patients with respect to healthy subjects, clearly indicating the involvement of these regulators in the onset and progression of AITD. The potential role of miRNAs in autoimmune thyroiditis is not an unexpected phenomenon, as miRNAs are well-established regulators of T cell activation, proliferation and cytokine production and are deregulated in several autoimmune diseases with both, anti- and pro-inflammatory functions [151,152]. In addition to identifying miRNAs differentially expressed in blood of GD and HT patients [153,154] or in thyroid tissues of individuals with different severity grades of AITDs [155], other studies strengthened the importance of miRNAs as markers of treatment response. miR-23b-5p and miR-92a were significantly increased in GD patients who achieved remission while a reduced level of let-7g-3p and miR-339-5p was associated with intractable GD patients [156]. Furthermore, increased miR-346 expression was shown as a possible predictive factor of relapse to antithyroid drug therapy for GD patients [157], and higher levels of five-signature miRNAs in serum were determined as highly independent patients [153,154] or in thyroid tissues of individuals with different severity grades of these diseases [155]. 

Other studies underlined the importance of miRNAs as markers of treatment response [158,159,160,161,162]. In fact, miR-23b-5p and miR-92a were significantly increased in GD patients who achieved remission, while a reduced level predictive markers for development of AITD with poor prognosis [158]. Studies focused not only on the detection of miRNA differences in AITD patients with respect to the normal controls but also on the modulation of their relative targets, allowed to unveil the biological significance of miRNAs deregulation and, consequently, their possible clinical implications in AITD. Next-generation sequencing analysis identified several miRNAs concordantly deregulated in thyroid tissues and serum from AITD patients: among these are miR-146a/b-5p, which displayed the most significant upregulation in thyroid tissues, miR-21-5p, miR-96-5p, miR-142-3p, miR-155-5p and others. Interestingly, the exploration of the gene ontology-enriched processes related to these molecules, showed association and cooperation with immunological system pathways such as cytokine signaling, Toll-like receptor cascade, interferon and B-cell receptor signaling [158]. Increase of miR-146 and decrease of miR-155 were implicated in the pathogenesis of Graves’ ophthalmopathy [159]. An integrative analysis of mRNA and miRNA array data reported that miR-146 and miR-155 were among miRNAs identified as deregulated also in Regulatory T (Treg) cells of GD. Tregs are a CD4 T cell subset critical for immunological homeostasis and tolerance to self-antigens [160]. The amount and/or function of Tregs are anomalous in patients with GD. Investigating the miRNAs altered in Tregs of GD patients and their putative target genes, which presented the expected modulation in the opposite direction, Wang and collaborators found a significant suppression of genes for retinoic acid pathway, protein ubiquitination and circadian rhythm pathways, suggesting the involvement of these pathways in GD Tregs abnormalities [161]. miR-346 may also participate in GD pathogenesis through the inhibition of follicular helper T (Tfh) cells, a subset of effector helper T cells crucial for the development of antigen-specific B cell immunity, by directly targeting Bcl-6, a positive regulator of Tfh cells [162]. 

A number of miRNAs involved also in the pathogenesis of HT disease have been described. Mir-142, which is highly expressed in HT patient serum, controlled the expression of Claudine (CLDN1), a tight junction protein downregulated in various autoimmune diseases [163]. Intracellular IL-10 expression was affected by the overexpression of Let7-e in PCMBs of HD [164], while downregulation of miR-125a-3p induced the upregulation of interleukin-23 receptor, a well-known strategic checkpoint in autoimmune diseases, in patients with HT [165]. 

Although for miRNAs, such as for methylation, a direct link among chemical exposure, miRNA alterations and AITD onset or progression has not yet been reported, some considerations can be noted. We have previously described the role played in thyroid tumor by the signaling pathway of NF-κB, a transcription factor that is affected by several toxicants and is activated in ATC wherein in turn it induces the overexpression of miR-146. NF-κB signaling pathway is also a major regulator of innate and adaptive immune responses [166]. Hence, it is tempting to assume that alterations of NF-κB pathway in T cells, as a possible consequence of pollutant exposure in susceptible individuals, could contribute to the deregulation of miR-146 that is implicated at different level in the pathogenesis of AITD. Several reports classified miR-146 in the group of miRNAs affected by EDCs such as BPA. Other miRNAs such as miR-21 and miR-19 were found to undergo modifications in AITD and were associated with immunological homeostasis [99,101]. miR-21 like miR-155 whose downregulation was correlated to GD, contain estrogen response elements in their promoters and therefore could be subject to estrogen regulation [167]. As BPA can act as an estrogen agonist, it has been suggested that BPA exposure possibly interfere with the expression of this class of miRNAs [168]. miRNA dysregulation by estrogen has been speculated in the reproductive system but it cannot be excluded that it is one of the possible causes of change in miR-155 expression observed in GD patients, especially considering that estrogen receptors are also expressed also in normal thyroid gland tissues.

## 5. Discussion 

Although direct and indirect evidence points to epigenetic changes as one of the likely mechanisms underlying the adverse health outcome of EDCs exposure, there is currently no clear link between EDC-induced epigenetic changes and a specific thyroid disease. Several reasons are at the root of this failure: first of all the limited number of studies that extensively investigate the consequence of exposures to environmental levels of EDCs in humans. Furthermore, experimental studies in animal models or in vitro studies on cell cultures to identify possible mechanisms of action for chemicals are sporadic and frequently give inconsistent results if compared with human studies. Other problems are related with the particular nature of the thyroid system. For definition, most EDCs can interfere with thyroid homeostasis, however the TH alterations possibly caused by EDC interference might appear insignificant compared to the wide reference range that characterizes TH levels. This can be of relevance particularly in small studies where the health consequences caused by even minor alterations in TH homeostasis risk being misinterpreted. Large studies are also necessary to deeply understand the impact of EDCs on epigenetics and, in turn, the influence of epigenetic mechanisms on endocrine function, keeping in mind that alterations of the thyroid homeostasis can be the other way around, affecting epigenetic signature [169]. 

In this review, starting from an analysis of the epigenetic modifications associated with a specific thyroid disease and from an overview of the EDCs implicated in triggering these diseases, we attempted to summarize the sparse experimental evidence supporting the involvement of epigenetic mechanisms in EDC-induced thyroid diseases. A limitation of this topic is that rarely are thyroid alteration caused by EDC exposure and epigenetic changes observed as a consequence of EDC exposure part of the same study or performed in the same cohort or even in the same kind of tissues or cells. However, some epigenetic mechanisms appear conserved in different EDC-triggered thyroid diseases. This is true not only for global modification such as DNA methylation that is deeply affected by EDC exposure, but also for more specific mechanisms. One example is miR-146, one of the most consistently upregulated miRNAs in PTC, FTC and ATC, which also displayed the most significant upregulation in thyroid tissues of GD patients. Other important signatures are represented by the overexpression of the maternally imprinted lncRNA H19 as a consequence of its demethylation in placental tissues, and the overexpression of this lncRNA in thyroid tumors wherein can act as an oncogene. These observations, suggesting a possible scenario where chemical exposure induces epigenetic modifications that can lead to different disease outcomes depending on the time and length of exposure and/or the genetic susceptibility, reinforce the importance of an integrated view of the relationship between EDC exposure, genetic and epigenetic mechanisms, and health alterations.

## 6. Conclusions

Overall, our review supports the hypothesis that epigenetic mechanisms could play an important role in determining thyroid diseases in response to environmental contaminants. A complex and often interconnected network of epigenetic modifications can be activated by a large number of widespread environmental contaminants. However, our analysis also clearly shows that further research in this field is still necessary to fully elucidate the relationships linking pollutants, epigenetic mechanisms and diseases pathogenesis and eventually to be able to exploit this knowledge for the development of new classes of diagnostic and prognostic disease tools.
